# RIDES: Robust Intrusion Detection System for IP-Based Ubiquitous Sensor Networks

**DOI:** 10.3390/s90503447

**Published:** 2009-05-11

**Authors:** Syed Obaid Amin, Muhammad Shoaib Siddiqui, Choong Seon Hong, Sungwon Lee

**Affiliations:** Department of Computer Engineering, School of Electronics and Information, Kyung Hee University, Korea; E-Mails: obaid@networking.khu.ac.kr (S.O.A.); shoaib@networking.khu.ac.kr (M.S.S.); drsungwon@khu.ac.kr (S.-W.L.)

**Keywords:** IP-USN, IDS, sensor networks, Bloom filter, CUSUM control charts

## Abstract

The IP-based Ubiquitous Sensor Network (IP-USN) is an effort to build the “Internet of things”. By utilizing IP for low power networks, we can benefit from existing well established tools and technologies of IP networks. Along with many other unresolved issues, securing IP-USN is of great concern for researchers so that future market satisfaction and demands can be met. Without proper security measures, both reactive and proactive, it is hard to envisage an IP-USN realm. In this paper we present a design of an IDS (Intrusion Detection System) called RIDES (Robust Intrusion DEtection System) for IP-USN. RIDES is a hybrid intrusion detection system, which incorporates both Signature and Anomaly based intrusion detection components. For signature based intrusion detection this paper only discusses the implementation of distributed pattern matching algorithm with the help of signature-code, a dynamically created attack-signature identifier. Other aspects, such as creation of rules are not discussed. On the other hand, for anomaly based detection we propose a scoring classifier based on the SPC (Statistical Process Control) technique called CUSUM charts. We also investigate the settings and their effects on the performance of related parameters for both of the components.

## 1. Introduction

The term “Ubiquitous Sensor Networks” (USN) is used to describe networks of smart sensor nodes capable of communicating wirelessly, and possessing limited computing power and storage capacity. USN can be used in a wide range of civilian and military fields, including environment and habitat monitoring, real-time healthcare, landmine detection, intelligent transport systems and so on [1]. Unfortunately, many of the current USN implementations use a proprietary suite of protocols which are specifically tailored for the environment under observation. Attaining interoperability among these various protocols is always desirable so that applications of one network paradigm can avail the services offered by other networks. Therefore, by empowering sensor nodes with IP (Internet Protocol) features we get a unified and simple naming and addressing hierarchy and consequently we obtain a certain level of interoperability among different sensor network standards. With the help of IP in sensor networks, we can also utilize the tools already available for configuring, managing, commissioning or accounting of the IP networks. Since the underlying protocols are based upon IP, designer of new sensor applications can use existing standards to speed up the design and development process. The network of IP enabled USN devices is usually termed as IP-USN (IP-based Ubiquitous Sensor Networks).

One of the promising features of IP-USN is remote accessibility of sensor nodes. This enables remote monitoring and management of sensitive environments such as healthcare systems. Health care systems are connected to patients and monitor patient's health, levels of medications and procedural outcomes. In such systems, it is not just sufficient to ensure confidentiality by encrypting the information sent out by the sensor nodes but it is also necessary to detect malicious or abnormal events in the system. Attacks and intrusions, for instance DoS (Denial of Service) or DDoS (Distributed DoS), against such systems may permit fatal damage to the health and safety of the patients. Such threats can be minimized by using firewalls and packet filtering. However, mechanisms that attempt to detect intrusions when occurred are also inevitable so that the intruder cannot damage the system for a long duration. This problem of detection is not specific to IP-USN. A wide variety of literature is available on intrusion detection for both IP and sensor networks. However, IP-USN devices supports broader range of applications, for example, a few of the implementations of IP-USN now have the support of embedded web services [2]. Any possible security holes in the implementation of such applications can be eliminated by updating the firmware of the device or by using any signature based IDS which knows about the pattern of the request required to exploit the bug. As updating firmware is not scalable, considering the large scale deployment of IP-USN, the later approach of signature-based IDS is an appealing solution.

Moreover, other attacks which exploit the weak hardware of the sensor networks are also possible in IP-USN. For example, IP layer usually works with available transport layer protocol. A few of the IP-USN implementations, such as Arch Rock [3], provide standard TCP and UDP protocols as transport layer for IP-USN; so that one can make connection easily to a sensor node to fetch the readings. Due to three way handshake protocol of TCP, a well known SYN flood attack can be launched on TCP-enabled sensor nodes. Likewise, other well known attacks such as Smurf [4] and UDP flooding [5] are also possible in IP-based sensor networks. Both of these types of attacks appear in the top 10 list of threats published by KrCERT [6]. None of these attack types have ever been addressed for sensor networks before. The question may arise here that why we cannot apply existing solutions of the aforementioned problems on IP-USN. It is so, because in IP-USN we have resource constrained devices and it is not an expedient decision to equip them with resource hungry intrusion detection schemes. Therefore, we need an IDS which is lightweight in terms of computation, communication and resources as well as able to detect new class of attacks possible in IP-USN environment.

In this paper we propose a design of an IDS for IP-USN environment called RIDES (Robust Intrusion DEtection System). RIDES is a hybrid IDS which incorporates both Signature based and Anomaly based IDS [7]. Thus, it is capable of detecting a large number of anomalies and intrusions, which makes RIDES a robust intrusion detection system. We preferred hybrid architecture due to the fact that there is a class of attacks which requires only a small number of packets to subvert the victim, such as Ping of Death [8], Land [8] and so on. In such cases, anomaly-based IDS fails drastically with high false negatives or Type-II errors. In other words, anomaly based IDS are unable to detect single packet attacks. Therefore, we strengthen our architecture with signature based attack detection. However, it is unwise to equip sensor nodes with the resource hungry detection schemes because signature-based intrusion detection system demands sufficient storage to store the signatures, and high processing power to match the incoming packets with stored signatures. To overcome this problem, we propose a novel coding scheme so that signature based IDS can be implemented on resource constrained sensor nodes. On the other hand, for anomaly-based IDS we need a scheme which is lightweight and capable of detecting even a minor shift from the normal behavior. Unfortunately, the later requirement is a major cause of large number of false positives or Type-I errors. To cope with these two contradictory requirements we adapt an optimal system from control theory and based our anomaly detection algorithm on CUSUM control charts. We also used the sensitivity of CUSUM to build a scoring based classifier. In short, we can summarize our contributions as follows:

- We accentuate the need of an IDS specifically tailored for IP-USN environment,
- Identify possible attack models in IP-USN environment,
- Introduce a dynamic creation of attack-signature identifier so that signature based IDS can be implemented on IP-USN,
- Design an anomaly based IDS for IP-USN environment,
- Provide evaluation results of both coding scheme and anomaly based IDS.

The rest of the paper is organized as follows: Section 2 discusses the background and relevant work of IP-USN. Section 3 gives the details of the proposed architecture and related schemes. Section 4 provides the evaluation results and finally Section 5 concludes our work.

## 2. Background and Relevant Work

### 2.1. IP-USN and Related Technologies

**Figure 1.**
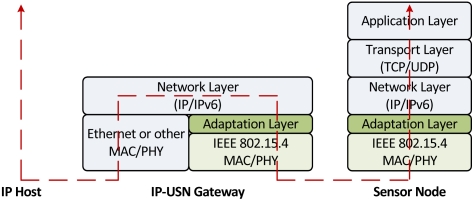
Traffic flow in IP-USN.

So far, 6LoWPAN (IPv6 over Low power WPAN) [9] is the only standard implementation of IP-USN. 6LoWPAN promises to transparently connect two different network paradigms, providing most of the advantages offered by the IP layer without forfeiting low-power operations of sensor networks. As 6LoWPAN is a merger of 802.15.4 and IPv6, it inherently supports 81 to 93 octets MTU (Maximum Transmission Unit), depending upon link layer security parameters. This MTU is significantly lower than the 1,280 octet MTU which is a minimum standard for IPv6. Therefore, an Adaptation Layer is used, as shown in Figure 1. The main function of the Adaptation Layer is to fragment and reassemble the packets. Figure 1 also depicts the position of gateway which is required to connect sensor nodes to the Internet. Devices in 6LoWPAN can be divided in to FFD (Full Function Device) and RFD (Reduced Function Device), depending upon their computation and memory resources. FFDs usually have more resources and can support RFDs by providing functions such as network coordination, packet forwarding, interfacing with other types of networks, etc. The IEEE 802.15.4 standard allows both star and peer-to-peer topologies with the presence of a central coordinator. Although, our IDS can be applied to any IP-based sensor networks, however throughout the paper we will refer 6LoWPAN to exemplify and illustrate the concept.

### 2.2. Signature Based Intrusion Detection

Intrusion Detection Systems can be divided broadly into two categories: Signature based Intrusion Detection and Anomaly based Intrusion Detection. In signature based IDS, if incoming packet header matches a certain set of rules, its payload is scrutinized against a set of known patterns (also called signatures). In case of a match, the ongoing activity is considered as an intrusion. Signature-based IDS is very efficient at sniffing out known attacks, however its efficiency is highly dependent on the stored signatures. As the number of patterns could range up to thousands, pattern matching consumes not only the storage but also the most of the CPU cycles to execute the complex pattern matching algorithms [10]. Therefore, so far there is no signature based IDS which can work on resource constrained sensor nodes. Our survey indicates that techniques like string matching, production rules, colored Petri nets, state transition diagrams, decision trees, and cluster structures have all been used to represent, store and match intrusion signatures [10-15]. Most of the commercial signature based IDS use automata theory or FSM (Finite State Machines) signature matching. Specially the works of Aho-Corasick [16] (its optimized version is used in Snort [17]) and Commentz-Walter [16] can be considered as epitomes in this area. However, these solutions are not practical for sensor networks due to their high demand of resources. In [10] authors presented a fast and scalable pattern matching scheme using Bloom filters [18]. This scheme scales well with multiple patterns matching and requires less storage and computation, which is discussed later. Therefore, we based our proposal on this scheme and proposed a coding mechanism so that a signature based IDS can be applied on low-power and low-storage sensor networks.

#### 2.2.1. Bloom Filter

**Figure 2.**
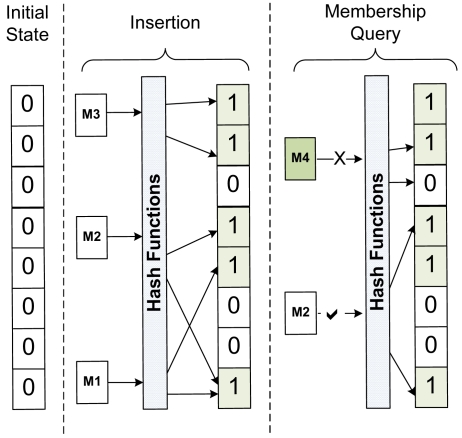
Basic operation of Bloom filters.

Bloom filter, proposed by Burton Bloom [18], is a simple space-efficient randomized data structure which, in its basic form, is represented by a bit array. The array is first initialized to all zeros. A Bloom filter computes *k* distinct digests for each entity with the help of independent uniform hash functions and uses the *r*-bit results to index into a *2^r^*-sized bit array. The indexed bits are then set to one. Figure 2 depicts the working of Bloom filters.

Membership queries can be carried out by computing the *k* digests on the incoming entities and checking the indicated bit positions. If any one of them is zero, the entity in question was not stored in the array. However, if all the bits are one, it is most likely that it was stored. A Bloom filter may yield a false positive, where it suggests that given entity is in Bloom filter even though it is not. However, the probability of false positives can be controlled, which is discussed later in the Evaluation section.

### 2.3. Anomaly Based Intrusion Detection

In an Anomaly-based Intrusion Detection certain parameters which reflect the normal state of a network system are defined or learned in advance. Any activity which then deviates from the predefined parameters significantly is considered as an intrusion [7]. Anomaly-based IDS is good at detecting new threats, but with certain number of false alarms, which is highly dependent on the parameters under observation and the definition of ‘significant change’. Methods from artificial intelligence, signal processing, statistics and data mining have been used to implement anomaly based IDS. Of these, schemes from the domain of signal processing, artificial intelligence and data mining are considered unsuitable for sensor networks as they rely on resource intensive algorithms for abnormality detection. In contrast, most of the statistical abnormality detection methods require basic sets of mathematical operations to detect the abnormality, which makes these schemes appealing for anomaly detection in IP-USN.

In the field of SPC (Statistical Process Control) techniques like Shewhart control charts, CUSUM control charts, and EWMA control charts are used for intrusion detection. A good survey of these techniques has been provided in [19]. Reference [19] also proposes modified forms of aforementioned control charts called MBM (Modified Batch Mean) charts to detect network intrusions. In [20] authors applied, tested, and compared two EWMA techniques to detect abnormality: EWMA for auto-correlated data and EWMA for uncorrelated data. Our scheme, on the other hand, uses CUSUM charts and utilizes the sensitivity of CUSUM charts to build a scoring classifier. We also investigate the settings and their affects on performance of certain parameters on our scheme.

## 3. RIDES Architecture

**Figure 3.**
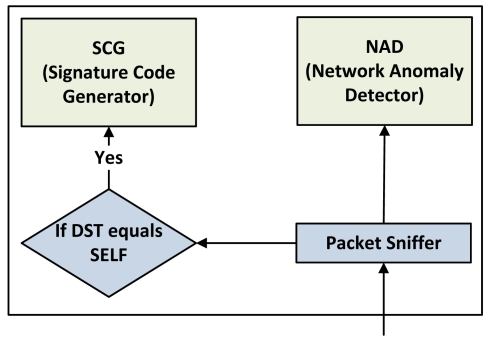
Internal architecture of intrusion detection component on a sensor node.

This section describes our intrusion detection framework for IP-USN. Table 1 shows the main components of RIDES and their placement:

Rules and signature database holds the complete attack-signature, signature-code and associated rules. Anomaly based Analyzer stores the CS (Contamination Score) of a network which will be discussed in detail in later section. Gateway or Sink is also responsible for initiating the intrusion response. However, intrusion response techniques such as traceback are out of the scope of this paper. Interested readers are encouraged to refer [21,22] for a good survey of intrusion response techniques. Figure 3 depicts the interaction between SCG (Signature-code Generator) and NAD (Network Anomaly Detector). As shown in Figure 3, a sensor node passes only those packets to SCG which are destined for itself. On the other hand, NAD checks every packet promiscuously. Figure 3 also depicts that both components are not connected to each other so that they can be realized separately on separate nodes as well. The rest of the section describes both of these components in detail.

### 3.1. SCG (Signature-code Generator)

In [10], authors presented a fast and lightweight pattern matching scheme for traditional IP networks using Bloom filters. However, in the current state of the technology this scheme cannot be implemented on sensor networks for the following reasons:

The scheme in [10] resides on a single machine and all traffic passes through that machine. Moreover, in [10] the authors used a separate hash table for Bloom filter match verification. Bloom filter match verification is required due to the probability of false positives associated with Bloom filters, as discussed later. However, in sensor networks many nodes are working as relay nodes. And due to resource limitations, it is unwise to place a hash table at every relaying node. In addition, if a distributed approach is used to implement this method such that the hash table and Bloom filters reside on separate machines (at the Sink and sensor node, respectively) then the entire payload of the packet would have to be sent to the Sink as well. This condition also lessens the efficiency of [10] because sensor needs more energy in transmission, as compared to calculation [23].

To overcome the aforementioned problems we propose the concept of a signature-code, a dynamically created attack-signature identifier which allows us to represent an attack-signature with only a few bytes. Therefore, instead of sending a complete payload we only send a signature-code for Bloom filter match verification to the Sink. Consequently, we not only save the storage of sensor nodes but also the energy required for transmitting long bit patterns. Thus, with the help of signature-code a lightweight signature based intrusion detection system can be realized.

#### 3.1.1. Signature-code

The signature-code is an r*⊖-bit number which is formed by concatenating the output of last *⊖* hash functions. Where *⊖* is the number of hash functions used to generate the signature-code. Let *S_i_* denotes the signature-code for attack-signature *i*. Then according to the definition, for *⊖* = *2 S_i_* can be given by:

(1) $$\begin{array}{l} S_{i}=H_{k-1}\left(i\right)⊙H_{k}\left(i\right)\\ \text{where}⊙\text{represents string concatination} \end{array}$$

**Figure 4.**
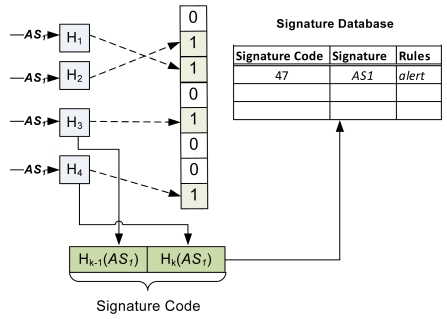
Generation of signature-code for the signatures having same length.

Figure 4 depicts generation of signature-codes. In Figure 4, the attack-signature *AS_1_* is passed through four hash functions which produce the *r-bit* output and set the corresponding bit to 1. For example, the output of hash function *H_3_* and *H_4_* is 100 and 111 (in terms of bits), respectively. Therefore, the bits on the 4^th^ and 7^th^ position (indexed from 0) are then set to 1. The outputs of the hash functions *H_3_* and *H_4_* are also used to generate the signature-code. In this case, the signature-code of *AS_1_* is 47.

We used signature-code to dynamically locate the attack-signatures and their associated rules in the signature database. It is possible that due to hash collision we may experience a signature-code collision. However, it is only possible when all of the hash functions involved in a signature-code generation experience a collision. Even if we have only one hash function which did not experience a hash collision, we would have a unique signature-code. For example, consider two input attack-signatures *a_1_* and *a_2_* then according to (1), without any hash collision the signature-codes would be:

$$\begin{array}{l} S_{a_{1}}=O_{1}⊙P_{1}\\ \text{where}\;O_{1}=H_{k-1}\left(a_{1}\right)\;\text{and}\;P_{1}=\;H_{k}\left(a_{1}\right)\\ S_{a_{2}}=O_{2}⊙P_{2}\\ \text{where}\;O_{2}=H_{k-1}\left(a_{2}\right)\;\text{and}\;P_{2}=H_{k}\left(a_{2}\right) \end{array}$$

Now suppose that we experience a hash collision in only one of the hash functions:

$$\begin{array}{cc} \text{If}\;(H_{k-1}(a_{1})=H_{k-1}(a_{2})=O_{1})\;\text{then}: & \text{If}(H_{k}(a_{1})=H_{k}(a_{2})=P_{1})\;\text{then}:\\ S_{a_{1}}=O_{1}⊙P_{1} & S_{a_{1}}=O_{1}⊙P_{1}\\ S_{a_{2}}=O_{1}⊙P_{2} & S_{a_{2}}=O_{2}⊙P_{1}\\ \text{If}(H_{k-1}(a_{1})=H_{k-1}(a_{2})=O_{2})\;\text{then}: & \text{If}(H_{k}(a_{1})=H_{k}(a_{2})=P_{2})\;\text{then}:\\ S_{a_{1}}=O_{2}⊙P_{1} & S_{a_{1}}=O_{1}⊙P_{2}\\ S_{a_{2}}=O_{2}⊙P_{2} & S_{a_{2}}=O_{2}⊙P_{2} \end{array}$$

It can be seen that signature-codes are unique, even if we have hash collision in one of the hash functions. Moreover, It is very unlikely that all of the hash functions experience a hash collision simultaneously for a given attack-signature; especially in a small data set with relatively large bit array *m*. It will be shown in the Evaluation section that with small dataset the signature-codes are completely unique.

#### 3.1.2. Working of SCG

The scheme in [10] uses separate Bloom filters for signatures of different sizes. Therefore, in the first phase, signatures of different length are passed from their respective Bloom filters. As a result, Bloom filters give us a bit array representation of the input signatures and signature-codes. The programmed Bloom filters are then placed at every sensor node. Sensor nodes pass the payload of each incoming packet through the Bloom filters. If a match is found, further processing over the packet is stopped and an alert signal is sent to the Sink, along with the signature-code, to verify the Bloom filter match. As there are separate Bloom filters for the signatures of different lengths, an alert signal might have multiple signature-codes within a packet. The signature-code points to the position of the signature and its associated rule in rules and signature database. The rules associated with the signature such as drop, notify, log etc. are then downloaded from the sink and applied on the packet in question. The scheme can be optimized by storing the downloaded rules in a small cache for later use; so that extra messaging can be avoided for similar packets. Since attack-signatures are rarely found in the packets, the swift check in Bloom filter avoids not only the unnecessary messaging but also the unneeded searching of attack-signature in whole signature database. Furthermore, the algorithm in [10] works for IP networks, where both signature creation and detection is performed on the same system. In such systems, false positives of Bloom filters are tolerable because in case of a false match we do not have to worry about messaging cost required to eliminate the compromised nodes. However, in sensor network, if we do not identify the attack-signature exactly then there would be a huge overhead of messaging to avoid the compromised nodes. Therefore, with the help of signature-code we try to identify exact attack-signatures so that extra messaging overhead can be minimized.

Sending of signature-code instead of entire payload also lessens the bits to be transmitted. This factor is mainly helpful in extending the lifetime of the sensor networks as shown in Evaluation section. In addition, the compressed representation of attack-signatures in a form of bit vectors also reduces the storage requirement on sensor nodes. Although this saving of space is inherited advantage of Bloom filters; however, application of Bloom filters in sensor network in the context of intrusion detection has never been addressed before.

In essence, this scheme is similar to the variant of Rabin-Karp algorithm provided in [24]. The complexity of Rabin-Karp multiple pattern algorithm can be given by *O(η* + *ε)*, where *η* is the number of strings stored in the Bloom filter, and *ε* is the number of patterns to be searched for a packet [24]. As the packet payload in IP-USN is much smaller than traditional networks [9], the given complexity is acceptable for IP-USN. For example, in 6LoWPAN, the payload is only 33 bytes for TCP traffic and 45 bytes for UDP traffic, without header compression techniques. If we consider the payload of Link layer security then we have only 21 bytes for TCP traffic and 33 bytes for UDP traffic [9]. With this limited payload we believe that the proposed scheme can work efficiently for IP-USN.

### 3.2. NAD (Network Anomaly Detector)

#### 3.2.1. CUSUM Control Charts

For anomaly based intrusion detection we used CUSUM control chart for detecting the abnormal network activity. In CUSUM charts a cumulative sum of the deviations of the sample values from a target value is observed. For example, let the samples of size *n* ≥ 1 are collected, and *X̄_j_* is the average of the *j^th^* sample. Then CUSUM can be plotted by:

(2) $$S_{i}=\sum_{j=1}^{i}\left({\bar{X}}_{j}-\mu_{0}\right)$$

Where *μ*_0_ is the in-control Mean of the system and *S_i_* is the cumulative sum up to and including the *i^th^* sample. If the value of CUSUM revolves around 0, the system is considered to be in-control. However, if the mean shifts to some value say *μ*_1_ such that *μ*_1_ > *μ*_0_, an upward or positive drift will be developed in the cumulative sum *S_i_*. On the other hand, if *μ*_1_ < *μ*_0_, a downward or negative drift in *S_i_* will be developed [25]. Therefore, if trend develops in the observed points either upward or downward, we can infer that system is in the abnormal state.

As *S_i_* reflects the information of several samples, cumulative sum charts are more effective than other types of control charts for detecting small process shifts. More importantly, CUSUM is more efficacious than other control charts for samples *n* = *1*. These two features make CUSUM a good candidate for the use in intrusion detection system. The former feature is particularly beneficial for sensor networks because in sensor networks even a minor shift in traffic parameters can be fatal. On the other hand, with sample size *n* = *1*, IDS can handle each new event notification discretely considering the sample size of 1 which provides us a way of online detection. In addition to that, usually sensor networks use event driven notification of the processes and events rarely follow any standard probability distribution model. Therefore, parametric approaches of change detection, which based upon the assumption of the normality of the data, are not efficient for network systems. However, non-parametric approaches of change detection such as CUSUM, due to its adaptable nature, provide very good and significant results. We also evaluated the non-parametric nature of CUSUM in Evaluation section by applying our scheme to two different traffic models.

#### 3.2.2. Detection Thresholds

The control limits of CUSUM charts can be defined by two methods 1) V-mask and 2) Tabular CUSUM. V-Mask is a visual procedure proposed by Barnard in 1959 [25]. It is rarely used to determine whether a process is out of control or not. More details of V-Mask can be found in [26]. On the other hand, Tabular CUSUM is an attractive approach if the CUSUM algorithm is implemented on computers [25]. To generate the tabular form, we use *H* and *K* parameters which are expressed in the original data units. Let *S_H_(i)* be an upper one-sided CUSUM for period *i* and *S_L_(i)* be a lower one-sided CUSUM for period *i*. These quantities can be calculated by:

(3) $$S_{H}(i)=\max[0,{\bar{x}}_{i}-(\mu_{0}+K)+S_{H}(i-1)]$$

(4) $$S_{L}(i)=\max[0,(\mu_{0}-K)-{\bar{x}}_{i}+S_{L}(i-1)]$$

where *S_H_(0)* and *S_L_(0)* are 0. When either *S_H_(i)* or *S_L_(i)* exceeds *H*, called decision interval, the process is out of control. *K* is called the reference value, which is calculated by equation (5). In equation (5), *μ*_1_ depicts the out-of-control mean value:

(5) $$\begin{array}{cc} K=\frac{\Delta }{2}, & \;\text{where}\Delta =\mu_{1}-\mu_{0} \end{array}$$

**Figure 5.**
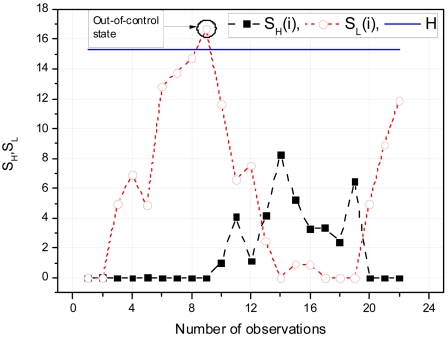
Example of CUSUM chart.

Figure 5 depicts an example CUSUM chart, made with the help of a tabular form. Control charts are usually evaluated by ARL (Average Run Length). If *p* is a probability that certain reading is not in control limits then the ARL of the system can be expressed as *1/p*. Studies suggest that for the 1 standard deviation shift, CUSUM shows good ARL properties for *H* = *ĥσ_X̄_* and *K* = *k̂σ_X̄_*, where *σ_X̄_* is the standard deviation of the sample variable used to create CUSUM control chart, (if *n*=1, *σ_X̄_* = *σ_X_*) [25]. The optimal value of *ĥ* is 4 or 5 and for *k̂* it is 0.4 or 0.5.

#### 3.2.3. Intrusion Detection and Overall Framework

In this paper we consider rate based DDoS attacks. Examples of such attacks possible in IP-based sensor networks are UDP flooding [5] and ICMP Smurf [4] attacks. Therefore, each detecting node maintains inter packet delay (delay between two consecutive packets). Initially each detecting node observes the network for a predefined time interval, which we termed as learning phase. In learning phase, each node calculates the values of process mean *μ*_0_, standard deviation *σ_X̄_*, and respective values of *H* and *K* for inter packet delay. After setting thresholds each detecting node starts to maintain the values of *S_h_* and *S_l_* for inter packet delay. If the value of *S_h_* or *S_l_* crosses the value of *H*, an attack signal is generated and sent to the IP-USN Gateway. However, as CUSUM charts are sensitive to a slight change, therefore, with small values of *k̂* many detecting nodes may generate a false alarm; more specifically a false positive. We utilized this behavior to create a scoring based CUSUM classifier. We termed the score of a classifier as CS (Contamination Score). CS of node *i* for *j^th^* learning phase can be represented by:

(6) $$CS_{i}=\max(\max({\text{F}}_{i}^{1}),\max({\text{F}}_{i}^{2}),\ldots ,\max({\text{F}}_{i}^{j}))$$

where, ${\text{F}}_{i}^{j}$ shows the false positive rate of CUSUM classifier in *j^th^* learning phase of node *i*. As the value of CS for a normal traffic is much lower than abnormal traffic, we can define a threshold above which the network is considered as contaminated. The maximum value of CS at a particular sensor node during learning phase is set as the threshold value for that node by the gateway. After receiving an alert signal from the sensor node, the gateway checks the ratio of alert signals against CS. If it is greater than CS the gateway initiates the attack response to subvert the attack or tries to identify the attacking or compromised nodes. Attack signal contains the source address of the victim, sensed data and the CUSUM value of the network parameters under observation. IP-USN Gateway, on the other hand, also manages the CUSUM of the sensed data. So that it can identify any false reading or malfunctioning sensor nodes. With the help of these network parameters we can detect a large set of cyber attacks ranging from traffic based DDoS attacks to false data injection.

### 3.3. Location of Intrusion Detection Components

There are usually two choices available for deciding the intrusion detection point in network systems, centralized or distributed. In a centralized system, a center node with high computing resources performs the intrusion detection based on the traffic profile gathered periodically from the distributed nodes. On the other hand, in a distributed IDS the detection algorithm runs on multiple nodes in the network. In case of attack, one or multiple nodes generate the attack signal which is passed to the centralized decision server. Both schemes can be characterized by the frequency of the generated messages. In case of centralized system the frequency of the generated messages to deliver the system state to the sink or base station is apparently higher than distributed systems. However, distributed IDS takes more network resources by duplicating the detection module on multiple nodes. Therefore, a balance is usually maintained by optimally choosing the right detection points. Fortunately in 6LoWPAN, the network topology allows us to optimally place the detecting nodes in the network. As discussed in Section 2, 6LoWPAN supports both star and mesh topology. In these, 6LoWPAN are expected to run more on mesh topology [9], in which FFDs are used as routers in scenarios where the receiver is not within direct reach of the sender of a packet. This makes FFD a good candidate for use as a packet interceptor.

There are also two advantages of implementing the scheme in distributed nature rather than having a single intrusion detection point at the gateway or the sink node. The first is that in an IP-USN network, all packets may not be destined to the gateway node; hence, internal attacks in the IP-USN would not be detected by the gateway node. The second reason is that by the collaboration of the relay nodes of the IP-USN for the intrusion detection, we are able to identify an intrusion packet earlier and might be able to stop the adversary packet from traversing through the network and wasting the scarce resources of the IP-USN.

## 4. Evaluation of RIDES

Table 2 lists the symbols used in the Evaluation section and their meanings:

### 4.1. Performance Evaluation of SCG

Currently, IP-USN is in its evolutionary stage. Thus, at the moment it is hard to realize any attack-signature in IP-USN traffic. Therefore, to evaluate the performance of our scheme we use Snort signature-set [17]. Snort can be regarded as a *de-facto* IDS for traditional IP networks. In current signature-set of version 2.8 of Snort [17], there are 13,339 attack-signatures. With current limited set of applications for IP-USN, this signature-set can easily be regarded as the upper bound for number of signatures in IP-based Ubiquitous Sensor Networks.

The performance of our signature based detection component mainly depends upon the size of the bit array *m*, the number of attack-signatures *n* and the number of hash functions *k*. We start our performance evaluation of the SCG from the analysis given in [27]. Given an array *m*, the probability of setting a certain bit to 1 is 1/m and the probability that is still *0* is 1 – 1/m. The probability that any of the hash function did not set the bit can be given by (1 – 1/m)^k^. If *n* numbers of attack-signatures are inserted, the probability that a certain bit is still unset can be given by (1 – 1/m)^kn^. And probability that it is set to 1 can be given by equation (7) [27]:

(7) $$1-{\left(1-\frac{1}{m}\right)}^{\text{kn}}$$

The probability that the *r* bit output of all *k* hash functions point to the locations in *m* which are already set is given by:

(8) $$\text{fpr}={\left(1-{\left(1-\frac{1}{m}\right)}^{\text{kn}}\right)}^{k}\approx {\left(1-e^{\frac{-\text{kn}}{m}}\right)}^{k}$$

Studies have shown that global minimum of false positive rate can be achieved with *k* = *ln2* × *(m/n)* [28]. In this case the right hand side of equation (8) becomes:

(9) $${\left(\frac{1}{2}\right)}^{k}={\left(0.6185\right)}^{\frac{m}{n}}$$

**Figure 6.**
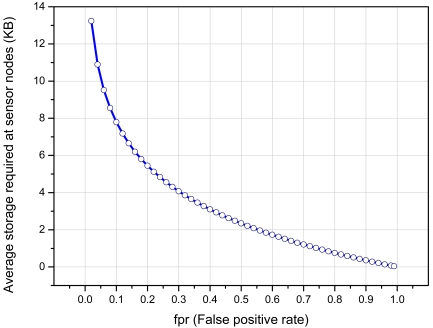
Size of *m* for representing current Snort signature-set with respect to *fpr*.

Further simplification shows that Bloom filter uses 1.441log_2_(1/*fpr*) bits of space per inserted attack-signature. As discussed above that there are 13,339 attack patterns available in the current release of Snort, so these attack signatures require approximately 252 KB of storage. Figure 6 shows the number of bits required to store current Snort signature-set by using our scheme. For example, with *fpr* = *0.024*, our scheme on an average requires 13 KB *(≈ 8bits/signature)* as compared to 252 KB to represent the whole signature-set. Approximately, we require *95*% less space than normal storage requirements.

If *ρ_i_* is the range of the hash function *H_i_* in bits then *R_i_* = 2*^ρi^* represents the number of unique signatures that can be indexed by the hash function *H_i_*. However, *R_i_* could be greater than *m*. Therefore, to limit it in the boundaries of bit vector *m*, we use a modulo operation. If *R_i_* ≤ *m* then the probability of hash collision *P_H_^i^* would be equal to *φ_i_*. Here *φ_i_* is the probability of the hash collision associated to individual hash functions without any modulo operation. However, for *R_i_* > *m* the *P_H_^i^* can be given by equation (10):

(10) $$p_{H^{i}}=1-\left(1-\varphi_{i}\right).{\left(1-\overset{1}{\;}/\underset{m}{\;}\right)}^{n}$$

**Figure 7.**
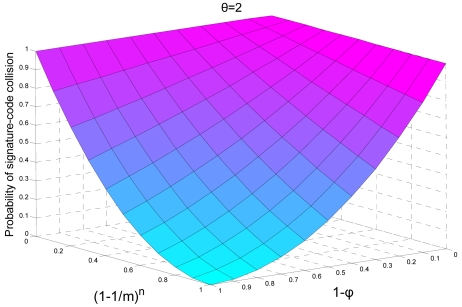
Probability of signature-code collision with respect to φ, m and n with ⊖ = 2.

As the signature-code collision occurs only when all of the hash functions, involved in the construction of signature-code, collide. Therefore, the probability of signature-code collision can be given by $p_{s}=\prod_{i=1}^{\theta }\left(p_{H^{k-i}}\right)$. Figure 7 depicts the effects of *φ, m* and *n* on signature-code for *⊖* = 2. We can see that as we increase the size of bit array *m* the probability of hash collision becomes low. On the other hand, as we increase the probability *φ_i_* the probability of hash collision approaches 1.

Furthermore, if *X* is the number of signatures added up to and including the first signature that experience a collision then the probability *P_X_(x)* of experiencing a collision in a signature-set can be given by:

(11) $$P_{X}(x)=p_{s}.{\left(1-p_{s}\right)}^{x-1}$$

And the expected number of signatures which can be added without experiencing a collision can be given by:

(12) $$\begin{array}{ll} E[X] & =\sum x.p_{S}{\left(1-p_{S}\right)}^{x-1}=p_{S}\sum x.{\left(1-p_{S}\right)}^{x-1}\\ & =p_{S}\sum x.\frac{{\left(1-p_{S}\right)}^{x}}{\left(1-p_{S}\right)}=\frac{p_{S}}{\left(1-p_{S}\right)}\sum x.{\left(1-p_{S}\right)}^{x}=\frac{p_{S}}{\left(1-p_{S}\right)}.\frac{\left(1-p_{S}\right)}{{\left(p_{S}\right)}^{2}}\\ & =>E[X]=\frac{1}{p_{S}} \end{array}$$

**Figure 8.**
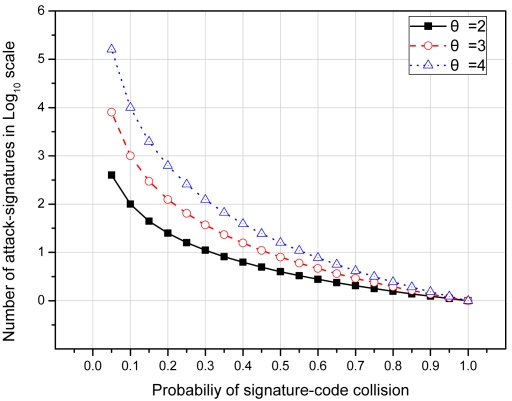
Number of signatures that can be added without experiencing a collision.

Figure 8 shows the expected number of signatures that can be added without experiencing a collision with increasing probability of signature-code collision and different values of *⊖* in *Log*_10_ scale. For example, for *p_H_^k^*^−1^ = *p_H_^k^*^−2^ = 0.01 and *⊖* = 2 the probability that signature-code will also experience a collision is 0.0001. Theoretically, this shows that approximately 10,000 signatures can be added without experiencing a collision. However, when implemented, we did not observe even a single signature-code collision in the 13,339 signature long signature-set of Snort [17].

**Figure 9.**
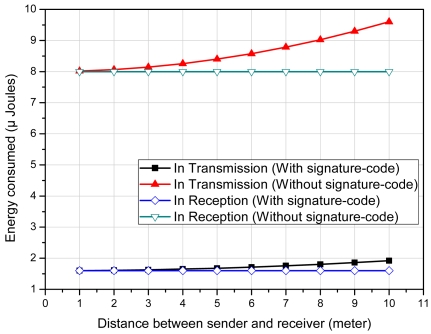
Comparison of energy consumption with and without signature-code.

The maximum length of an attack-signature in current Snort's signature-set is 487 bytes; whereas, the average length of an attack-signature is 20 bytes. If we assume that the incoming packet only contains attack-signature of the length of 20 bytes then without our scheme 20 bytes are required to be sent to the Sink for searching a rule in signature database. On the other hand, our scheme requires only 4 bytes with *⊖* = 2 to search a rule in signature database, even for the biggest signature in Snort signature-set. Figure 9 shows the differences in energy consumption in single-hop transmission and reception of a message in both of these schemes according to the radio model given in [23]; with average attack-signature length of 20 bytes, *⊖* = 2 and *r* = 16.

### 4.2. Performance Evaluation of NAD

We use UDP flooding to evaluate the performance of NAD. We conducted our simulation on ns-2 [29]. We placed 25 nodes randomly in mesh topology. Source – destination pairs are chosen randomly and the selected source initiates the data transfer at random time for random duration. Other parameters of the simulation are given in Table 3.

**Figure 10.**
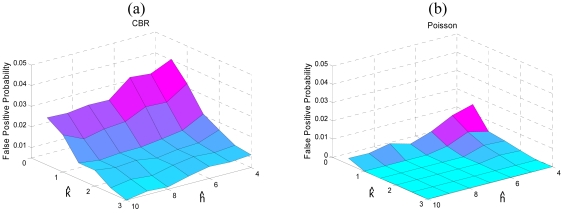
(a) False positive probability for different values of *ĥ* and *k̂* for CBR traffic; (b) False positive probability for different values of *ĥ* and *k̂* for Poisson traffic.

We made two modules for our Anomaly detection system. The first module is used to learn the parameters *μ*_0_ and *σ_X_*; while the second module is used to detect the intrusion in the network. The value of *μ*_0_ is used in equation (3) and (4); whereas the value of *σ_X_* is used to derive the value of *H* and *K* as discussed in Section 3.2.2. To verify the recommended values of *ĥ* and *k̂* given in Section 3.2.2 we first learn the parameters *μ*_0_ and *σ_X_* from one simulation and then apply it on the same simulation for different values of *ĥ* and *k̂*. In this way we nullify the effects of traffic dissimilarity and only consider the changes of *ĥ* and *k̂*. Figure 10a,b shows the false positive probability with different values of *ĥ* and *k̂* for two different traffic models. As shown in the figure, a little increment in *k̂* significantly reduces the *FPP*. On the other hand, the values of *ĥ* slightly affects the *FPP*. This also shows that CUSUM charts are more sensitive to the value of *k̂* instead of *ĥ*. However, as discussed later, higher values of *k̂* turn CUSUM into a bad classifier. In other words, with higher values of *k̂* CUSUM is unable to classify normal traffic and abnormal traffic.

**Figure 11.**
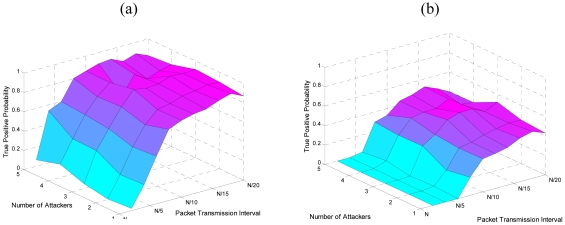
(a) True positive probability with increasing number of attackers and increasing intensity of the attack with *k̂* = 0.5. (b)True positive probability with increasing number of attackers and increasing intensity of the attack with *k̂* = 1.

Our next set of simulations depicts the performance of CUSUM with increasing traffic load and number of attackers. Figures 11a,b shows the true positive probability with *k̂* = 0.5 and *k̂* = 1, respectively. For both of the simulations we use *ĥ* = 5. In these figures, *N* represents the normal packet transmission interval. As we can see that with *k̂* = 0.5 the CUSUM is very sensitive and able to detect even minor surge in the traffic load. While with *k̂* = 1 we are able to detect the attack if the inter-packet transmission interval is at least five times more than the normal rate.

To further evaluate the performance of our scoring based classifier we used ROC (Receiver Operating Characteristics) curves [30]. ROC curve provides a useful way for organizing classifiers and visualizing their performance. For intrusion detection system, ROC curve summarizes the relationship between two of the most important characteristics: *FPP* (False Positive Probability) and *TPP* (True Positive Probability). To compare the performance of classifiers we usually use a scalar quantity called AUC (Area Under the ROC curve). The value of AUC will always be between 0 and 1. Classifiers with high AUC values have better performance than the classifiers with low AUC values [30].

**Figure 12.**
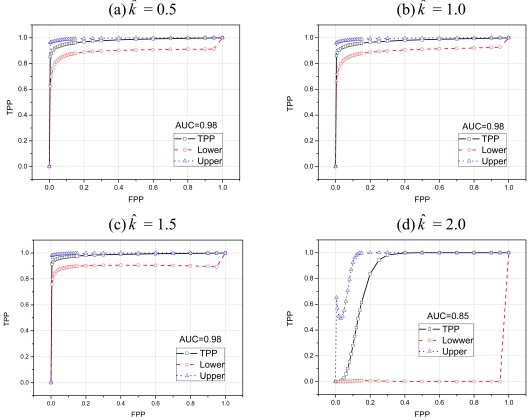
ROC curves when attacking traffic rate is 10 times faster than normal traffic rate.

**Figure 13.**
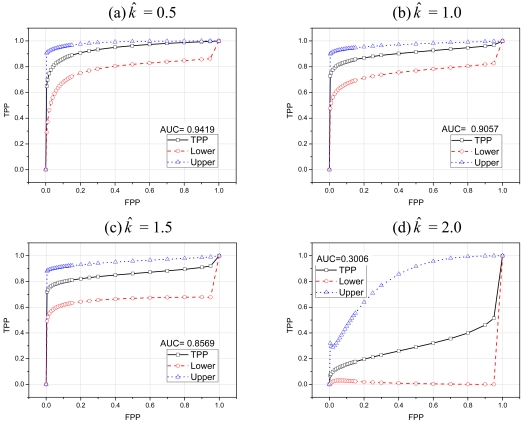
ROC curves when attacking traffic rate is 5 times faster than normal traffic rate.

Figures 12a,b,c and d show the ROC curves with different values of *k̂*. This set of ROC curves depicts the classification performance of our scheme when the attacking packets are 10 times faster than normal packets. Therefore, our scoring based classifier easily classifies abnormal traffic from normal traffic, as shown by AUC values. Figures 13a,b,c and d depict the classification performance of our scheme when the attacking packets are only five times faster than normal packets. As we can see that even with little difference between normal and attacking traffic our scheme classifies most of the attacks with acceptable false positive probability. However, as we can see in Figures 12d and 13d that with *k̂* = 2 our scoring scheme performs very badly in classification. This difference is more prominent especially in Figure 13d, where the difference between normal and attacking traffic is less. In fact, any real life classifier doesn't have AUC less than 0.5. This behavior is due to the high reference value *k̂* of CUSUM charts, which severely affects the performance of CUSUM and consequently of our scoring classifier. In both Figure 12 and 13 the lower and upper curves depict the 95% confidence interval boundaries [30].

All of the above simulation results show that with *ĥ* = 5 and *k̂* = 0.5 we got a good balance between *FPP* and *TPP* for our scoring based classifier, irrespective of the traffic model under consideration. It is so, because the dissimilarity of traffic pattern is reflected in *σ_X_*, which makes our scheme adaptable for different traffic models.

## 5. Conclusions

In this paper, we have provided the details of a specifically tailored IDS (Intrusion Detection System) for IP-USN (IP-based Ubiquitous Sensor Networks), called RIDES (Robust Intrusion DEtection System). To the best of our knowledge, RIDES is the first intrusion detection system for any IP-based sensor network. RIDES can be categorized under hybrid IDS, which has both Anomaly and Signature based intrusion detection components.

For signature based intrusion detection component, this paper only discussed the implementation of a distributed pattern matching algorithm with the help of signature-code, a dynamically created attack-signature identifier. Other aspects like rule creation are not discussed. Evaluation results show that we require approximately 95% less space than normal storage requirements to store Snort signature-set. In addition, with the help of signature-codes we can greatly enhance the lifetime of a sensor node.

On the other hand, for Anomaly based intrusion detection component, we utilized a lightweight statistical process control method called CUSUM control charts and proposed a scoring based classifier to mitigate the sensitivity of CUSUM. We also discussed the effects of different parameters of CUSUM on our scheme. We evaluated the performance of our scoring classifier with the help of ROC curves. ROC curves show that our scoring classifier can effectively classify normal traffic and abnormal traffic and the probability of false positives is also very low. These results conclude that RIDES is appropriate for resource constraint sensor nodes for detecting network intrusions.

Finally, intrusion detection alone is not sufficient; effective response to detected intrusion is equally important. Therefore, currently we are exploring the automated intrusion response schemes such as traceback or packet filtering that should be activated after detecting a network intrusion.

## Figures and Tables

**Table 1.** Main Components of RIDES and Their Placement.

Gateway/Sink	Rules and Signatures Database
	Anomaly based Analyzer
IP-based sensor nodes	SCG (Signature-code Generator), Bloom filters
	NAD (Network Anomaly Detector)

**Table 2.** Symbols and Their Meanings.

***Symbols***	**Meaning**
*m*	Size of Bloom filter's bit array
*n*	Number of attack-signatures to be added
*k*	Number of hash functions
*ρ_i_*	Range of the hash function *H_i_* in bits
*r*	Controlled range of the hash functions in bits
*⊖*	Number of hash functions used in signature-code generation
*fpr*	False positive rate of Bloom filters
*p_H_^i^*	Probability of experiencing a collision in hash function *H^i^*
*p_S_*	Probability of signature-code collision
*P_X_(x)*	Probability of experiencing a collision in a signature-set
*K*	Reference value of CUSUM chart
*H*	Decision interval of CUSUM chart
*FPP*	False positive probability of scoring classifier
*TPP*	True positive probability of scoring classifier

**Table 3.** Simulation Parameters of NAD.

**Parameter**	**Value**
Number of nodes	25
Number of data generating nodes	11
Terrain	50 m × 50 m
Topology	Mesh
Traffic model	cbr/poisson
Beaconing mode	No
Maximum data rate	250 kbps
Routing protocol	AODV
MAC protocol	802.15.4

## References

[1] Choe Y.H., Kelly T., Adolph M. (2008). Ubiquitous Sensor Networks.

[2] Culler D. (2007). Embedded Web Services: Making Sense out of Diverse Sensors.

[3] Hui J., Culler D. (2008). Extending IP to low-power, wireless personal area networks. IEEE Internet Comput..

[4] Garber L. (2000). Denial-of-service attacks rip the Internet. Computer.

[5] CERT Advisory CA-1996-01 UDP Port Denial-of-Service Attack.

[6] Korea Internet Security Center (In Korean).

[7] Axelsson S. (2000). Intrusion detection systems: A survey and taxonomy.

[8] Kendall K. (1999). A database of computer attacks for the evaluation of intrusion detection systems. Ph.D. dissertation.

[9] Montenegro G., Kushalnagar N., Hui J., Culler D. (2007). IPv6 over Low-Power Wireless Personal Area Networks (6LoWPANs): Overview, assumptions, problem statement and goals (RFC 4919).

[10] Dharmapurikar S., Lockwood J. (2006). Fast and scalable pattern matching for network intrusion detection systems. IEEE JSAC.

[11] Anderson D., Frivold T., Valdes A. (1995). Next-generation intrusion detection expert system (NIDES): A summary; Tech. Rep. SRI-CSL-97-07.

[12] Vigna G., Eckmann S., Kemmerer R. (2000). The STAT tool suite.

[13] Kumar S. (1995). Classification and detection of computer intrusions. Ph.D. dissertation.

[14] Lee W, Stolfo S.J., Mok K. (1999). Mining in a data-flow environment: Experience in network intrusion detection.

[15] Ye N., Li X. (2001). A scalable clustering technique for intrusion signature recognition.

[16] Stephen D.L. (1994). String Searching Algorithms; In Lectures Notes Series on Computing.

[17] Snort – the de facto standard for intrusion detection/prevention.

[18] Bloom B. (1970). Space/time trade-offs in hash coding with allowable errors. ACM Commun..

[19] Park Y. (2005). A statistical process control approach for network intrusion detection. Ph.D. dissertation.

[20] Ye N, Vilbert S, Chen Q. (2003). Computer intrusion detection through EWMA for autocorrelated and uncorrelated data. IEEE Trans. Reliab..

[21] Stakhanova N., Basu S., Wong J. (2007). A taxonomy of intrusion response systems. Int. J. Inf. Comput. Secur..

[22] Amin S.O., Siddiqui M.S., Hong C.S. (2008). A novel IPv6 traceback architecture using COPS protocol. Ann. Telecommun..

[23] Heinzelman W.R., Chandrakasan A., Balakrishnan H. (2000). Energy-efficient communication protocol for wireless microsensor networks.

[24] Rabin-Karp string search algorithm.

[25] Montgomery D.C., Runger G.C. (2004). Applied Statistics and Probability for Engineers.

[26] Montgomery D.C. (2001). Introduction to Statistical Quality Control.

[27] Broder A., Mitzenmacher M. (2004). Network applications of bloom filters: A survey. Internet Math..

[28] Fan L., Cao P., Almeida J., Broder A. (2000). Summary cache: A scalable wide-area web cache sharing protocol. IEEE/ACM TON.

[29] McCanne S., Floyd S., Fall K., Varadhan K. Network Simulator – ns-2.

[30] Fawcett T. (2006). An introduction to ROC analysis. Pattern Recognition Lett..

